# Supplementary material for: Acute aerobic exercise intensity does not modulate pain potentially due to differences in fitness levels and sex effects: results from a pharmacological fMRI study
Source: eLife. 2025 Aug 6;14:RP102392. doi: 10.7554/eLife.102392 (PMC12327945; doi:10.7554/eLife.102392)
Supplement: Supplementary file 1. [file elife-102392-supp1.docx]

Supplementary File 1a. Functional Threshold Power Test Protocol.

| Phase | Duration | Description | Cadence (RPM) | Details |
| --- | --- | --- | --- | --- |
| Warm-Up | 20-min | Endurance Pace | 65-70 |  |
| Intervals | 3 x 1-min | Fast pedalling | 90-100 | 1-min recovery between intervals |
| Recovery | 5-min | Active Recovery | 65 |  |
| Time-Trial | 5-min | Maximum Effort | 65-75 | Maximum effort that can be maintained constantly for 5 minutes in a steady state |
| Recovery | 10-min | Active Recovery | 65 |  |
| FTP Test | 20-min | Maximum Effort | 65 - 75 | Maximum effort that can be maintained constantly for 20 minutes in a steady state |
| Cool Down | 10-min | Active Recovery | 65 |  |
| FTP = Functional Threshold Power; RPM = revolutions per minute | | | | |

Supplementary File 1b. *Χ^2^-*test to test for differences in the distribution of menstrual cycle phases (*n* = 17 female participants).

|  | *Χ^2^* | *df* | *P* |
| --- | --- | --- | --- |
| Experimental Day 2 | 0.21 | 3 | 0.98 |
| Experimental Day 3 | 0.77 | 3 | 0.86 |
| Exp Day 2 vs. Exp Day 3 | 15.80 | 9 | 0.07 |
| *Χ^2^-*test to test for differences in the distribution of menstrual cycle phases (follicular, ovulatory, and luteal phase) for female participants on both experimental days separately and between experimental days (*n* = 17). *N* = 4 female participants were on hormonal contraceptives and were not included in this analysis.*df* = degrees of freedom. | | | |

Supplementary File 1c. Full LMER model output of parametric effect on behavioural heat pain ratings in saline condition.

| Fixed Effects | Estimate | *SE* | *df* | *t* | *P* |
| --- | --- | --- | --- | --- | --- |
| Intercept | -34.33 | 2.77 | 70 | -12.40 | <2×10^-16^ |
| Stimulus Intensity | 1.42 | 0.03 | 1358 | 51.84 | <2×10^-16^ |
| Treatment_order | 10.22 | 3.51 | 37 | 2.91 | 0.006 |
| LMER = linear mixed effects model, *SE* = standard error, *df* = degrees of freedom. Subject and number of pain ratings were included as random effects. | | | | | |

Supplementary File 1d. Post-hoc paired *t*-tests (Tukey adj.) for LMER model parametric effect saline behavioural heat pain ratings with according effect size (Cohens *d*).

| Contrast | Estimate | *SE* | *df* | *t-ratio* | *P* | *Cohens \|d\|* |
| --- | --- | --- | --- | --- | --- | --- |
| VAS 30 – VAS 50 | -20.4 | 1.07 | 1359 | -18.99 | < 0.001 | 1.25 |
| VAS 30 – VAS 70 | -57.0 | 1.07 | 1359 | -53.24 | < 0.001 | 3.49 |
| VAS 50 – VAS 70 | -36.6 | 1.07 | 1359 | -34.16 | < 0.001 | 2.37 |
| LMER = linear mixed effect model, *SE* = standard error, *df* = degrees of freedom | | | | | | |

Supplementary File 1e. Full LMER model output of stimulus intensity and drug on behavioural heat pain ratings.

| Fixed Effects | Estimate | *SE* | *df* | *t* | *P* |
| --- | --- | --- | --- | --- | --- |
| Intercept | -31.18 | 2.68 | 80 | -10.89 | <2×10^-16^ |
| Stimulus Intensity | 1.43 | 0.03 | 2757 | 49.43 | <2×10^-16^ |
| Drug | -0.37 | 2.14 | 2755 | -0.17 | 0.86 |
| Treatment_order | 2.82 | 3.48 | 37 | 0.81 | 0.42 |
| Stimulus Intensity: drug | 0.10 | 0.04 | 2755 | 2.46 | 0.01 |
| LMER = linear mixed effects model, *SE* = standard error, *df* = degrees of freedom. Subject and number of pain ratings were included as random effects. | | | | | |

Supplementary File 1f. Post-hoc paired *t*-tests (Tukey adj.) for LMER model interaction stimulus intensity and drug on heat pain ratings.

| Contrast | Estimate | *SE* | *df* | *t-ratio* | *P* | *Cohens \|d\|* |
| --- | --- | --- | --- | --- | --- | --- |
| SAL 30 – NLX 30 | -2.22 | 1.13 | 2753 | -1.97 | 0.36 | 0.13 |
| SAL 30 – SAL 50 | -20.51 | 1.13 | 2755 | -18.10 | < 0.001 | 1.19 |
| SAL 30 – NLX 50 | -25.98 | 1.13 | 2755 | -22.94 | < 0.001 | 1.50 |
| SAL 30 – SAL 70 | -57.04 | 1.13 | 2755 | -50.40 | < 0.001 | 3.30 |
| SAL 30 – NLX 70 | -63.27 | 1.13 | 2755 | -55.91 | < 0.001 | 3.66 |
| NLX 30 – SAL 50 | -18.29 | 1.13 | 2755 | -16.13 | < 0.001 | 1.06 |
| NLX 30 – NLX 50 | -23.76 | 1.13 | 2755 | -20.98 | < 0.001 | 1.38 |
| NLX 30 – SAL 70 | -54.81 | 1.13 | 2755 | -48.44 | < 0.001 | 3.17 |
| NLX 30 – NLX 70 | -61.04 | 1.13 | 2755 | -53.95 | < 0.001 | 3.53 |
| SAL 50 – NLX 50 | -5.47 | 1.13 | 2753 | -4.84 | < 0.001 | 0.32 |
| SAL 50 – SAL 70 | -36.52 | 1.13 | 2755 | -32.24 | < 0.001 | 2.11 |
| SAL 50 – NLX 70 | -42.76 | 1.13 | 2755 | -37.74 | < 0.001 | 2.47 |
| NLX 50 – SAL 70 | -31.05 | 1.13 | 2755 | -27.43 | < 0.001 | 1.80 |
| NLX 50 – NLX 70 | -37.28 | 1.13 | 2755 | -32.93 | < 0.001 | 2.16 |
| SAL 70 – NLX 70 | -6.23 | 1.13 | 2753 | -5.52 | < 0.001 | 0.36 |
| LMER = linear mixed effect model, SAL = saline, NLX = naloxone, *SE* = standard error, *df* = degrees of freedom. | | | | | | |

Supplementary File 1g. Full LMER model output of stimulus intensity on behavioural differential heat pain ratings [NLX – SAL].

| Fixed Effects | Estimate | *SE* | *df* | *t* | *P* |
| --- | --- | --- | --- | --- | --- |
| Intercept | 6.10 | 2.87 | 100.36 | 2.13 | 0.04 |
| Stimulus Intensity | 0.10 | 0.04 | 77 | 2.46 | 0.02 |
| Treatment_order | -14.80 | 3.07 | 37 | -4.83 | 2.4×10^-5^ |
| LMER = linear mixed effects model, *SE* = standard error, *df* = degrees of freedom, NLX = naloxone, SAL = saline. Subject and number of pain ratings were included as random effects. | | | | | |

Supplementary File 1h. Post-hoc paired *t*-tests (Tukey adj.) for LMER model interaction stimulus intensity and drug on heat pain ratings for females.

| Contrast | Estimate | *SE* | *df* | *t-ratio* | *P* | *Cohens \|d\|* |
| --- | --- | --- | --- | --- | --- | --- |
| SAL 30 – NLX 30 | 0.16 | 1.57 | 1478 | 0.10 | 1.00 | 0.01 |
| SAL 30 – SAL 50 | -18.71 | 1.57 | 1478 | -11.93 | < 0.001 | 1.06 |
| SAL 30 – NLX 50 | -21.46 | 1.57 | 1478 | -13.67 | < 0.001 | 1.22 |
| SAL 30 – SAL 70 | -53.28 | 1.57 | 1478 | -33.99 | < 0.001 | 3.03 |
| SAL 30 – NLX 70 | -59.93 | 1.57 | 1478 | -38.23 | < 0.001 | 3.41 |
| NLX 30 – SAL 50 | -18.87 | 1.57 | 1478 | -12.03 | < 0.001 | 1.07 |
| NLX 30 – NLX 50 | -21.61 | 1.57 | 1478 | -13.77 | < 0.001 | 1.23 |
| NLX 30 – SAL 70 | -53.44 | 1.57 | 1478 | -34.09 | < 0.001 | 3.04 |
| NLX 30 – NLX 70 | -60.09 | 1.57 | 1478 | -38.34 | < 0.001 | 3.42 |
| SAL 50 – NLX 50 | -2.74 | 1.57 | 1478 | -1.75 | 0.50 | 0.16 |
| SAL 50 – SAL 70 | -34.56 | 1.57 | 1478 | -22.01 | < 0.001 | 1.97 |
| SAL 50 – NLX 70 | -41.22 | 1.57 | 1478 | -26.25 | < 0.001 | 2.35 |
| NLX 50 – SAL 70 | -31.82 | 1.57 | 1478 | -20.27 | < 0.001 | 1.81 |
| NLX 50 – NLX 70 | -38.48 | 1.57 | 1478 | -24.50 | < 0.001 | 2.19 |
| SAL 70 – NLX 70 | -6.66 | 1.57 | 1478 | -4.25 | 0.003 | 0.38 |
| LMER = linear mixed effect model, SAL = saline, NLX = naloxone, *SE* = standard error, *df* = degrees of freedom. | | | | | | |

Supplementary File 1i. Post-hoc paired *t*-tests (Tukey adj.) for LMER model interaction stimulus intensity and drug on heat pain ratings for males.

| Contrast | Estimate | *SE* | *df* | *t-ratio* | *P* | *Cohens \|d\|* |
| --- | --- | --- | --- | --- | --- | --- |
| SAL 30 – NLX 30 | -5.00 | 1.62 | 1262 | -3.10 | 0.02 | 0.03 |
| SAL 30 – SAL 50 | -22.46 | 1.63 | 1262 | -13.79 | < 0.001 | 1.34 |
| SAL 30 – NLX 50 | -31.13 | 1.63 | 1262 | -19.14 | < 0.001 | 1.85 |
| SAL 30 – SAL 70 | -61.34 | 1.62 | 1262 | -37.90 | < 0.001 | 3.65 |
| SAL 30 – NLX 70 | -67.08 | 1.62 | 1262 | -41.45 | < 0.001 | 4.00 |
| NLX 30 – SAL 50 | -17.45 | 1.63 | 1262 | -10.72 | < 0.001 | 1.04 |
| NLX 30 – NLX 50 | -26.13 | 1.63 | 1262 | -16.06 | < 0.001 | 1.56 |
| NLX 30 – SAL 70 | -56.33 | 1.62 | 1262 | -34.81 | < 0.001 | 3.36 |
| NLX 30 – NLX 70 | -62.07 | 1.62 | 1262 | -38.35 | < 0.001 | 3.70 |
| SAL 50 – NLX 50 | -8.68 | 1.62 | 1262 | -5.35 | < 0.001 | 0.52 |
| SAL 50 – SAL 70 | -38.88 | 1.62 | 1262 | -23.94 | < 0.001 | 2.32 |
| SAL 50 – NLX 70 | -44.62 | 1.62 | 1262 | -27.47 | < 0.001 | 2.66 |
| NLX 50 – SAL 70 | -30.20 | 1.62 | 1262 | -18.62 | < 0.001 | 1.80 |
| NLX 50 – NLX 70 | -35.94 | 1.62 | 1262 | -22.16 | < 0.001 | 2.14 |
| SAL 70 – NLX 70 | -5.74 | 1.62 | 1262 | -3.55 | 0.005 | 0.34 |
| LMER = linear mixed effect model, SAL = saline, NLX = naloxone, *SE* = standard error, *df* = degrees of freedom. | | | | | | |

Supplementary File 1j. Full LMER model output of stimulus intensity and sex on behavioural differential heat pain ratings [NLX – SAL].

| Fixed Effects | Estimate | *SE* | *df* | *t* | *P* |
| --- | --- | --- | --- | --- | --- |
| Intercept | 0.93 | 3.66 | 104.79 | 0.25 | 0.80 |
| Stimulus Intensity | 0.17 | 0.05 | 76 | 3.12 | 0.003 |
| Sex | 11.26 | 5.03 | 111.12 | 2.24 | 0.03 |
| Treatment_order | -14.85 | 3.05 | 36 | -4.88 | 2.19×10^-5^ |
| Stimulus Intensity: Sex | -0.15 | 0.08 | 76 | -1.89 | 0.06 |
| LMER = linear mixed effects model, *SE* = standard error, *df* = degrees of freedom, NLX = naloxone, SAL = saline. Subject and number of pain ratings were included as random effects. | | | | | |

Supplementary File 1k. Full LMER output of exercise intensity on heat pain ratings in the saline condition.

| Fixed Effects | Estimate | *SE* | *df* | *t* | *P* |
| --- | --- | --- | --- | --- | --- |
| Intercept | 36.29 | 2.46 | 53.75 | 14.75 | 2×10^-16^ |
| Exercise Intensity | 1.19 | 1.55 | 1354 | 0.77 | 0.44 |
| Treatment_order | 10.244 | 3.51 | 37 | 2.91 | 0.006 |
| LMER = linear mixed effects model, *SE* = standard error, *df* = degrees of freedom. Subject and number of pain ratings were included as random effects. | | | | | |

Supplementary File 1l. Full LMER output of exercise intensity on betas extracted from ROI RVM in the saline condition.

| Fixed Effects | Estimate | *SE* | *df* | *t* | *P* |
| --- | --- | --- | --- | --- | --- |
| Intercept | 0.07 | 0.10 | 49.73 | 0.72 | 0.48 |
| Exercise Intensity | 0.05 | 0.07 | 194 | 0.63 | 0.53 |
| Treatment_order | 0.23 | 0.12 | 37 | 1.96 | 0.06 |
| LMER = linear mixed effects model, *SE* = standard error, *df* = degrees of freedom. Subject was included as a random effect. | | | | | |

Supplementary File 1m. Full LMER output of exercise intensity on betas extracted from ROI PAG in the saline condition.

| Fixed Effects | Estimate | *SE* | *df* | *t* | *P* |
| --- | --- | --- | --- | --- | --- |
| Intercept | 0.09 | 0.09 | 51.62 | 0.93 | 0.36 |
| Exercise Intensity | 0.02 | 0.08 | 194 | 0.31 | 0.76 |
| Treatment_order | 0.11 | 0.12 | 37 | 0.96 | 0.35 |
| LMER = linear mixed effects model, *SE* = standard error, *df* = degrees of freedom. Subject was included as a random effect. | | | | | |

Supplementary File 1n. Full LMER output of exercise intensity on betas extracted from ROI Frontal Midline in the saline condition.

| Fixed Effects | Estimate | *SE* | *df* | *t* | *P* |
| --- | --- | --- | --- | --- | --- |
| Intercept | -0.16 | 0.09 | 46.61 | -1.67 | 0.10 |
| Exercise Intensity | -0.08 | 0.06 | 194 | -1.26 | 0.21 |
| Treatment_order | 0.19 | 0.12 | 37 | 1.65 | 0.11 |
| LMER = linear mixed effects model, *SE* = standard error, *df* = degrees of freedom. Subject was included as a random effect. | | | | | |

| Fixed Effects | Estimate | *SE* | *df* | *t* | *P* |
| --- | --- | --- | --- | --- | --- |
| Intercept | 36.52 | 3.00 | 54.3 | 15.23 | 2×10^-16^ |
| Exercise Intensity | 1.19 | 1.60 | 2755 | 0.75 | 0.45 |
| Drug | 4.50 | 1.60 | 2755 | 2.81 | 0.005 |
| Treatment_order | 2.82 | 3.48 | 37 | 0.81 | 0.42 |
| Exercise Intensity x Drug | 0.27 | 2.27 | 2755 | 0.12 | 0.91 |
| LMER = linear mixed effects model, *SE* = standard error, *df* = degrees of freedom. Subject and number of pain ratings were included as random effects. | | | | | |

Supplementary File 1o. Full LMER output of exercise intensity and drug treatment on heat pain ratings.

Supplementary File 1p. Full LMER output of exercise intensity and drug treatment on betas extracted from ROI RVM.

| Fixed Effects | Estimate | *SE* | *df* | *t* | *P* |
| --- | --- | --- | --- | --- | --- |
| Intercept | 0.04 | 0.09 | 44.63 | 0.49 | 0.63 |
| Exercise Intensity | 0.06 | 0.05 | 426 | 1.05 | 0.29 |
| Drug | -0.02 | 0.04 | 426 | -0.15 | 0.68 |
| Treatment_order | 0.25 | 0.12 | 37 | 2.15 | 0.04 |
| Exercise Intensity x Drug | 0.01 | 0.05 | 426 | 0.23 | 0.82 |
| LMER = linear mixed effects model, *SE* = standard error, *df* = degrees of freedom. Subject was included as a random effect. | | | | | |

Supplementary File 1q. Full LMER output of exercise intensity and drug treatment on betas extracted from ROI PAG.

| Fixed Effects | Estimate | *SE* | *df* | *t* | *P* |
| --- | --- | --- | --- | --- | --- |
| Intercept | 0.13 | 0.08 | 48.45 | 1.68 | 0.10 |
| Exercise Intensity | 0.08 | 0.06 | 426 | 1.42 | 0.16 |
| Drug | 0.05 | 0.04 | 426 | 1.17 | 0.24 |
| Treatment_order | 0.11 | 0.10 | 37 | 1.18 | 0.25 |
| Exercise Intensity x Drug | 0.06 | 0.06 | 426 | 1.00 | 0.32 |
| LMER = linear mixed effects model, *SE* = standard error, *df* = degrees of freedom. Subject was included as a random effect. | | | | | |

Supplementary File 1r. Full LMER output of exercise intensity and drug treatment on betas extracted from ROI frontal midline.

| Fixed Effects | Estimate | *SE* | *df* | *t* | *P* |
| --- | --- | --- | --- | --- | --- |
| Intercept | -0.15 | 0.07 | 46.44 | -2.16 | 0.04 |
| Exercise Intensity | -0.05 | 0.04 | 426 | -1.12 | 0.26 |
| Drug | -0.03 | 0.03 | 426 | .1.09 | 0.28 |
| Treatment_order | 0.12 | 0.09 | 37 | 1.36 | 0.18 |
| Exercise Intensity x Drug | 0.03 | 0.04 | 426 | 0.63 | 0.53 |
| LMER = linear mixed effects model, *SE* = standard error, *df* = degrees of freedom. Subject was included as a random effect. | | | | | |

Supplementary File 1s. Full linear model output from the model including FTP on difference score heat pain ratings (LI – HI exercise) in the saline condition.

| Fixed Effects | Estimate | *SE* | *t* | *P* |  |
| --- | --- | --- | --- | --- | --- |
| Intercept | -10.84 | 4.66 | -2.33 | 0.03 |  |
| FTP | 6.45 | 2.56 | 2.52 | 0.02 |  |
| Treatment_order | -4.33 | 2.49 | -1.74 | 0.09 |  |
| FTP = functional threshold power (weight-corrected), *SE* = standard error, *df* = degrees of freedom. Subject and number of pain ratings were included as random effects. | | | | | |

Supplementary File 1t. LMER output from the model including FTP, drug, and sex as fixed effects on differential heat pain ratings (LI exercise – HI exercise).

| Fixed Effects | Estimate | *SE* | *df* | *t* | *P* |
| --- | --- | --- | --- | --- | --- |
| intercept | -1.52 | 6.09 | 67.27 | -0.25 | 0.80 |
| Stimulus intensity | -0.05 | 0.03 | 190 | -1.35 | 0.18 |
| FTP | 2.62 | 3.61 | 56.77 | 0.73 | 0.47 |
| Sex | -24.28 | 10.56 | 57 | -2.30 | 0.03 |
| Drug | -9.76 | 5.72 | 190 | -1.71 | 0.09 |
| Treatment_order | -4.72 | 2.09 | 34 | -2.26 | 0.03 |
| Sex: Drug | 28.61 | 10.34 | 190 | 2.77 | 0.006 |
| FTP: Drug | 4.81 | 3.48 | 190 | 1.39 | 0.17 |
| FTP: Sex | 11.98 | 5.54 | 57.61 | 2.16 | 0.03 |
| FTP: Sex: Drug | -13.12 | 5.41 | 190 | -2.43 | 0.016 |
| LMER = linear mixed effects model, FTP = functional threshold power (weight-corrected), *SE* = standard error, *df* = degrees of freedom. The subject was included as a random effect. | | | | | |

Supplementary File 1u. Participant characteristics.

|  | Overall Mean (SD) | Females Mean (SD) | Males Mean (SD) |
| --- | --- | --- | --- |
| *N* | 39 | 21 | 18 |
| Age (years) | 26.03 (4.83) | 25.33 (5.10) | 26.83 (4.35) |
| Weight (kg) | 70.95 (12.14) | 63.33 (7.53) | 79.83 (10.38) |
| Height (cm) | 177.10 (9.08) | 170.52 (6.35) | 184.78 (4.58) |
| BMI | 22.51 (2.64) | 21.80 (2.59) | 23.33 (2.53) |
| FTP (Watt/kg) | 1.79 (0.48) | 1.58 (0.44) | 2.03 (0.40) |
| Training Volume (h/w) | 4.44 (3.24) | 3.88 (2.63) | 5.08 (3.81) |
| SD = Standard deviation. kg = kilogram. cm = centimeter. h/w = hours per week. | | | |

Supplementary File 1v. POMS Mood Ratings (Wilcoxon signed-rank test).

|  | *W* | *P* |
| --- | --- | --- |
| Naloxone Pre - Post | | |
| Fatigue | 544.5 | < 0.001 |
| Drive | 174 | 0.01 |
| Discontent | 22 | 0.05 |
| Dejection | 10.5 | 0.001 |
| Saline Pre - Post | | |
| Fatigue | 381.5 | 0.28 |
| Drive | 409.5 | 0.39 |
| Discontent | 12 | 0.23 |
| Dejection | 33.5 | 0.007 |
| Saline Post – Naloxone Post | | |
| Fatigue | 226 | 0.15 |
| Drive | 437 | 0.19 |
| Discontent | 17 | 0.67 |
| Dejection | 57.5 | 0.42 |
| *W* = test statistic. |  |  |

Supplementary File 1w. Side Effects Naloxone (Wilcoxon signed-rank test).

|  | *W* | *P* |
| --- | --- | --- |
| Lethargy | 91 | 0.61 |
| Dry Mouth | 77.5 | 0.06 |
| Dry Skin | 68 | 0.45 |
| Blurred Vision | 25.5 | 0.29 |
| Dizziness | 26 | 0.37 |
| Headache | 6 | 0.37 |
| Sickness | 4 | 0.41 |
| *W* = test statistic. | | |

Supplementary File 1x. Small Volume Correction (SVC) mask for pain modulation effects based on preregistered midbrain ROIS.

| Label | Description | Atlas/Source | x | y | z | Sphere (mm) |
| --- | --- | --- | --- | --- | --- | --- |
| PAG | Periaqueductal grey | Brainstem Navigator (Bianciardi et al., 2018, 2015; García-Gomar et al., 2022, 2019; Singh et al., 2021, 2020) | - | - | - | - |
| RVM | Rostral ventral medulla | ROI from Tinnermann et al. (2017) (Tinnermann et al., 2017) | 0 | -32 | -44 | 3 |
| Frontal Midline |  |  |  |  |  |  |
|  | pgACC bilateral | From Vega et al., (2016) (Vega et al., 2016) | - | - | - | - |
|  | vmPFC | From Vega et al., (2016) (Vega et al., 2016) | - | - | - | - |
| xyz-coordinates are provided in the MNI space. mm = millimetre, PAG = Periaqueductal gray, pgACC = pregenual anterior cingulate cortex, RVM = Rostral ventral medulla, vmPFC = ventromedial prefrontal cortex. | | | | | | |
